# Leveraging Centralized Health System Data Management and Large Language Model–Based Data Preprocessing to Identify Predictors for Radiation Therapy Interruption

**DOI:** 10.1200/CCI-25-00218

**Published:** 2025-10-28

**Authors:** Fekede Asefa Kumsa, Christopher L. Brett, Soheil Hashtarkhani, Rezaur Rashid, Lokesh Chinthala, Janet A. Zink, Robert L. Davis, Arash Shaban-Nejad, David L. Schwartz

**Affiliations:** ^1^ Center for Biomedical Informatics, Department of Pediatrics, College of Medicine, University of Tennessee Health Science Center, Memphis, TN; ^2^ University of Tennessee Graduate School of Medicine, Knoxville, TN; ^3^ Department of Radiation Oncology, College of Medicine, University of Tennessee Health Science Center, Memphis, TN; ^4^ Department of Preventive Medicine, College of Medicine, University of Tennessee Health Science Center, Memphis, TN

## Abstract

**PURPOSE:**

Unplanned treatment interruptions represent an important care quality shortfall for patients undergoing cancer radiotherapy. This study aimed to evaluate use of a centralized electronic health record warehouse and large language model–based data preprocessing to facilitate identification of risk factors for radiation therapy interruptions (RTI).

**METHODS:**

We analyzed demographic, behavioral, clinical, and neighborhood-level data for 2,130 patients treated with radiotherapy at the University of Tennessee Medical Center in Knoxville. Treatment interruptions were measured as missed days, adjusted for weekends and holidays. Multinomial logistic regression was used to identify factors associated with moderate (2-4 days) and severe (≥5 days) RTI.

**RESULTS:**

Moderate RTI occurred in 15.8% of patients, while 7.7% experienced severe RTI. Moderate delays were associated with genitourinary cancer (adjusted odds ratio (AOR), 3.81; 95% CI, 1.24 to 11.66), prostate cancer (AOR, 2.44; 95% CI, 1.34 to 4.46), and Medicaid coverage (AOR, 2.22; 95% CI, 1.32 to 3.73). Severe RTI was associated with marital status (AOR for divorced or separated patients, 1.86; 95% CI, 1.18 to 2.94), head and neck cancer (AOR, 2.31; 95% CI, 1.10 to 4.87), gynecologic cancer (AOR, 2.97; 95% CI, 1.30 to 6.79), Medicaid insurance (AOR, 3.43; 95% CI, 1.77 to 6.64), daily dose of ≤225 cGy (AOR, 2.55; 95% CI, 1.21 to 5.37), and a total dose of ≥6,000 cGy (AOR, 2.30; 95% CI, 1.09 to 4.88). Severe interruptions were also significantly associated with high neighborhood social vulnerability (AOR, 2.60; 95% CI, 1.32 to 5.09).

**CONCLUSION:**

Automated data preprocessing permitted efficient identification of treatment course length, marital status, disease site, Medicaid coverage, and socially vulnerable locations as significant factors associated with RTI. These findings underscore the need for data-driven risk assessment and intervention strategies to maintain cancer treatment quality at scale.

## INTRODUCTION

Radiation therapy is a cornerstone of modern oncology and contributes to the care of more than half of all patients with cancer. However, efficacy relies upon rigorous adherence to daily therapy, with unplanned interruptions being strongly associated with increased rates of treatment failure.^1-6^ Unfortunately, treatment interruptions remain common.^4,7-9^ To limit consequences of missed treatment, it is essential to identify and remediate clinical and social risks contributing to preventable radiation therapy interruptions (RTI). Mechanistic causes for RTI are multifactorial. Logistical challenges related to treatment scheduling and machine downtime frequently contribute to delays.^4-6^ Patient-specific factors, including poor performance status, comorbidities, and treatment-related toxicities, can also lead to treatment noncompliance and unplanned hospitalizations.^10^ Patients from socially disadvantaged backgrounds or limited financial resources are also disproportionately affected. Research in urban settings has identified that minority race, Medicaid or uninsured status, and even Medicare coverage in older populations are associated with a significantly higher risk of RTI.^11-14^

**CONTEXT**

**Key Objective**
How can a centralized electronic health record warehouse and large language model (LLM)–based data preprocessing facilitate identification of risk factors for radiation therapy interruption (RTI)?
**Knowledge Generated**
Nearly one quarter of radiotherapy patients in our Appalachian catchment area miss two or more scheduled treatment days, and about 10% miss a week or more. LLM models, GPT-4.0 and BioBERT, achieved over 90% accuracy for International Classification of Diseases–coded diagnoses and over 81% accuracy for free-text entries, with classifications validated by oncology experts.
**Relevance *(J.L. Warner)***
This study is interesting in several ways. First, it demonstrates that RTI can be defined and measured across a broad catchment area (the state of Tennessee). Second, it reveals a number of independent factors associated with RTI, which could lead to targeted interventions. Future integration of chemotherapy exposure would be quite informative for RTI in the context of combined modality therapy.*
*Relevance section written by *JCO: Clinical Cancer Informatics* Editor-in-Chief Jeremy L. Warner, MD, MS, FAMIA, FASCO.

This study is a continuation of our group's previously published work in the Upper Mississippi Delta region.^11^ Here, we leverage a centralized health system electronic health data repository to optimize identification of clinical and socioeconomic determinates of elevated risk for RTI events in a socially and geographically distinct metro-rural region located within the same US state (Knoxville, TN area in Central Appalachia) to guide potential downstream risk-triage strategies and intervention design, which could be scaled across different areas and populations.

## METHODS

### Data Source

We obtained individual-level demographic, behavioral, and clinical data for patients with cancer from a state-wide, multi-institutional research Enterprise Data Warehouse (rEDW) managed by members of our group. The rEDW is a standardized aggregated health care data warehouse comprising 4.6 million unique patients in a single, comprehensive, integrated repository of clinical and research data. Data are contributed from multiple health care organizations associated with the University of Tennessee Health Science Center. Analysis for this report was limited exclusively to data from the University of Tennessee Medical Center (UTMC, Knoxville, TN).

### Ethics

This study was reviewed and approved by the Institutional Review Board (IRB) of the University of Tennessee Health Science Center for analysis without personal identifiers (IRB grant number: 16-04888-XP).

### Variables

Individual-level data extracted from the rEDW included demographics (age, race, and marital status), behavioral factors (smoking status, substance use, and alcohol use), and clinical data (International Classification of Diseases [ICD] codes, insurance type, treatment dates, daily and total radiation fraction size, and treatment intent). Social determinants of health (SDH) were assessed at the census tract level and included the Social Vulnerability Index (SVI) from the CDC (2022), and driving distance (in miles) between the patient's residence and the treatment center. Driving distance in miles between the patient's residence and the treatment center was calculated using ArcGIS software, on the basis of the road network, the centroid of the patient's residential census tract, and the geocoded location of the treatment facility. The SVI ranks geographic areas on the basis of 16 social factors, grouped into four categories: socioeconomic status, household characteristics, minority status, and housing type and transportation.^15^

### Study Outcome Measures

The primary outcome of this study was the number of missed radiation treatment days. Missed treatment days were calculated on the basis of the anticipated versus actual completion dates of therapy, adjusted for weekends and federal holidays. We categorized this outcome at the patient level as one of three groups: no missed days (missed 0-1 scheduled sessions), moderate delays (2-4 days missed), and severe delays (five or more days missed).

### Data Preprocessing

The data were cleaned and assessed for completeness using simple frequency analyses and cross-tabulations. Missing data and outliers were excluded from the data set. Missed treatment days were calculated, and the distance between each participant's residence and their radiotherapy treatment center was determined using the GEOID (a unique identifier for US Census geographic areas) of the patient's residential census tract to estimate driving distance to the treatment facility. Specific data elements from patient electronic health records were unstructured and required preprocessing to be used in the model. The health insurance carrier field contained over 1,000 unique entries with varying formats and payer names, which were standardized into predefined general categories. Similarly, for the cancer diagnosis field, ICD, 10th Revision (ICD-10) codes were used if available; otherwise, cancer types were extracted from free-text clinical notes.

To process these unstructured data, the GPT-4 model was used as an application protocol interface in Python. A tailored prompt and examples were provided to categorize the information. The results were manually validated by clinical oncologists (D.L.S. and C.L.B.) to ensure accuracy and reliability. Methods and validation outcomes have been previously described.^16^

### Data Analysis

To account for the possibility that certain risk factors may differentially influence minor (2-4 days) versus excessive (5 or more days) treatment interruptions, we conducted bivariable and multivariable multinomial logistic regression analyses. All variables hypothesized to be associated with RTI were included in the final multivariable model. The crude odds ratio and adjusted odds ratio (AOR), along with 95% CIs, were calculated to measure the strength of association between the outcome and independent variables. Model fit was assessed using the likelihood ratio chi-square (LR χ^2^) test. The LR χ^2^ statistic, with 94 df, was 286.79 (*P* < .001), indicating that the final model with all variables significantly improves the explanation of the observed data compared with the reduced model. To reduce the risk of overfitting, we used a careful variable selection process, prioritizing model simplicity by excluding highly correlated variables. Multicollinearity was evaluated using variance inflation factors (VIF), with a VIF score of 1.84, suggesting no significant collinearity among the factors. Variables with a *P*-value of <.05 in the multivariable analysis were considered significantly associated with missing a day of radiation treatment. All statistical analyses were performed using STATA version 18.

Additionally, to examine the spatial distribution of RTI rates, we used patients' census tract IDs to aggregate data and generate a ZIP code–level proportional symbol map. These rates were then overlaid with the ZIP code–level distribution of SVI scores to visually assess potential spatial patterns. All geographic visualizations and statistical analyses were performed using ArcGIS Pro, enabling the identification of ZIP code areas with significantly higher RTI rates and their potential linkage to community-level vulnerability.

## RESULTS

### Study Cohort

**FIG 1.**
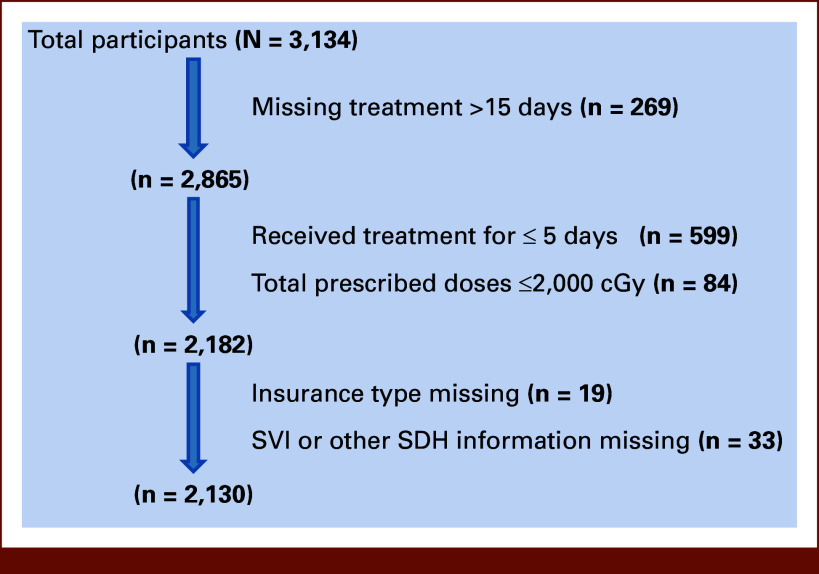
Flowchart of study cohort selection. SDH, social determinants of health; SVI, Social Vulnerability Index.

A total of 3,134 consecutive patients underwent radiation treatment between August 2020 and May 2024. Of these, 269 patients were excluded from analysis because of missing treatment days exceeding 15 days, since such an extensive delay likely indicated treatment discontinuation. Additionally, 599 patients who received a treatment course lasting ≤5 days and 84 patients who received a total treatment dose ≤2,000 cGy were excluded, as these patients were presumed to have undergone short-course palliation or specialized stereotactic treatment, both of which are considered to carry a very low risk of interruption. Furthermore, 19 patients who were missing insurance-related information and 33 patients lacking neighborhoods-level information were excluded. A final total of 2,130 patients were included in the analysis (Fig 1). Study cohort characteristics are summarized in Table 1.

**TABLE 1.** Clinical and Sociodemographic Characteristics of the Study Cohort

Variable	No. (%)
Age, years	
<50	262 (12.3)
50-65	787 (36.9)
>65	1,081 (50.8)
Race	
White	1,996 (93.7)
Black or others	134 (6.3)
Marital status	
Single	363 (17.0)
Married	1,213 (57.0)
Separated/divorced	382 (17.9)
Unknown	172 (8.1)
Sex	
Female	1,136 (53.3)
Male	994 (46.4)
Smoking status	
Current smoker	202 (9.5)
Former smoker	374 (17.5)
Never	466 (21.9)
Unknown	1,088 (51.1)
Substance use	
Current	57 (2.7)
Never	1,166 (54.7)
Past	42 (2.0)
Unknown	865 (40.6)
Alcohol use	
Current	501 (23.5)
Never	677 (31.8)
Past	110 (5.2)
Unknown	842 (39.5)
Primary disease site	
Benign	45 (2.1)
Breast	504 (23.7)
CNS	94 (4.4)
GI	185 (8.7)
Genitourinary	28 (1.3)
Gynecologic	114 (5.4)
Head and neck	273 (12.8)
Hematologic	53 (2.5)
Lung/thoracic	232 (10.9)
Metastasis	188 (8.8)
Prostate	272 (12.8)
Skin	44 (2.1)
Soft tissue	32 (1.5)
Unknown	66 (3.0)
Insurance carrier	
Clinical trial	21 (1.0)
Dual Medicare/Medicaid	65 (3.0)
Employment/individual/commercial	843 (39.6)
Medicaid	124 (5.8)
Medicare	716 (33.6)
Military	61 (2.9)
No insurance	119 (5.6)
Other/unknown	181 (8.5)
Treatment intent	
Palliative	342 (16.0)
Curative	1,738 (81.6)
Unknown	50 (2.4)
Distance to radiotherapy facility (miles)	
0.00-5.00	255 (12.0)
5.01-25.00	1,338 (62.8)
>25.00	537 (25.2)
Daily prescribed fraction size, cGy	
≤225	1,397 (62.0)
>225	733 (38.0)
Total prescribed dose, cGy	
<5,000	556 (26.1)
5,000-5,999	648 (30.3)
≥6,000	925 (43.6)
No. of prescribed days	
<25	808 (37.9)
≥25	1,322 (62.1)
Social vulnerability level at census tract level	
Very low	631 (29.6)
Low	682 (32.0)
Moderate	577 (27.1)
High	240 (11.3)

About half of the patients (1,081, 50.8%) were older than 65 years. The majority were White (1,996, 93.7%). A total of 1,213 patients (57.0%) were married, 202 (9.5%) were current smokers, and 374 (17.6%) were former smokers. Additionally, 57 patients (2.7%) reported current substance use, and 501 (23.5%) reported current alcohol use. The most common diagnoses were breast (504, 23.7%), head and neck (273, 12.8%), prostate (272, 12.8%), and thoracic cancers (232, 10.9%). The largest share of patients was covered by employment-based plans or private payers (843, 39.6%), while 716 (33.6%) were enrolled in Medicare and 124 (5.8%) were insured by Medicaid. One thousand seven hundred thirty-eight patients (81.6%) received therapy with curative intent, and 537 (25.2%) patients resided more than 25 miles from the radiotherapy treatment center. The total prescribed dose ranged from 2000 to 7920 cGy, with 928 (43.6%) prescribed a total dose of more than 6,000 cGy. Seven hundred thirty-three patients (34.4%) received hypofractionated treatment, defined as a dose of more than 225 cGy per day. A total of 1,322 (62%) patients received treatment for more than 25 days.

A significant proportion of the patients resided in census tracts with a high (240, 11.3%) or moderate (577, 27%) social vulnerability level.

### Univariate Analysis of RTI Risk Associations

Before excluding cases with missing or outlier data, 76.4% missed 0-1 day, 16.6% missed 2-4 days, and 7.0% of patients missed 5 or more days of treatment. After excluding these cases, 1,632 patents (76.6%; 95% CI, 74.8% to 78.4%) missed 0-1 days of treatment. Three hundred thirty-five patients (15.7%; 95% CI, 14.2% to 17.3%) missed 2-4 days (moderate RTI), while 163 patients (7.7%; 95% CI, 6.5% to 8.8%) missed 5 or more days (severe RTI; *P* < .001). Patient and disease-related variables with statically significant correlation with severity of RTI included marital status (*P* = .011), sex (*P* = .004), smoking status (*P* < .001), substance use (*P* = .012), primary disease site (*P* < .001), insurance carrier (*P* < .001), and treatment intent (*P* < .001). Treatment-related variables found to be statistically significant included daily fraction size, total treatment dose, and treatment course length (*P* < .001 for all variables, Table 2).

**TABLE 2.** Univariate Analysis of Radiation Therapy Interruption Severity

Variable	Radiation Therapy Interruption Severity, No. (%)			*P*
	No Interruption (0-1 day)	Minor Interruption, % (2-4 RT Breaks)	Major Interruption, % (5+ RT Breaks)	
Total, N = 2,130	1,632	335 (15.7)	163 (7.7)	
Age, years				.374
<50	211 (12.9)	31 (9.3)	20 (12.3)	
50-65	602 (36.9)	122 (36.4)	63 (38.7)	
>65	819 (50.2)	169 (53.3)	80 (49.0)	
Race				.724
White	1,533 (93.9)	312 (93.1)	151 (92.6)	
Black or others	99 (6.1)	23 (6.9)	12 (7.4)	
Marital status				**.011**
Single	274 (16.8)	58 (17.3)	31 (19.0)	
Married	958 (58.7)	178 (53.1)	77 (47.2)	
Separated/divorced	268 (16.4)	70 (20.9)	44 (27.0)	
Unknown	132 (8.1)	29 (8.7)	11 (6.8)	
Sex				**.004**
Female	908 (55.3)	155 (46.3)	78 (47.9)	
Male	729 (44.7)	180 (53.7)	85 (52.1)	
Smoking status				**<.001**
Current smoker	123 (7.5)	50 (14.9)	29 (17.8)	
Former smoker	282 (17.3)	63 (18.8)	29 (17.8)	
Never	370 (22.7)	66 (19.7)	30 (18.4)	
Unknown	857 (52.5)	156 (46.6)	75 (46.0)	
Substance use				**.012**
Current	36 (2.21)	12 (3.58)	9 (5.52)	
Never	899 (55.09)	177 (52.84)	90 (55.21)	
Past	25 (1.53)	13 (3.88)	4 (2.45)	
Unknown	672 (41.18)	133 (39.70)	60 (36.81)	
Alcohol use				.630
Current	385 (23.6)	80 (23.9)	36 (22.1)	
Never	508 (31.1)	106 (31.6)	63 (38.6)	
Past	83 (5.1)	19 (5.7)	8 (4.9)	
Unknown	656 (40.2)	130 (38.8)	56 (34.4)	
Primary disease site				**<.001**
Benign	35 (2.1)	7 (2.0)	3 (1.9)	
Breast	424 (26.0)	60 (17.9)	20 (12.3)	
CNS	82 (5.0)	9 (2.7)	3 (1.9)	
GI	141 (8.5)	27 (8.1)	17 (10.4)	
Genitourinary	12 (0.7)	6 (1.8)	10 (6.1)	
Gynecologic	82 (5.3)	14 (4.2)	18 (11.0)	
Head and neck	188 (11.5)	48 (14.3)	37 (22.7)	
Hematologic	48 (3.0)	5 (1.5)	0 (0.00)	
Lung/thoracic	167 (10.2)	49 (14.6)	16 (9.8)	
Metastasis	160 (9.8)	22 (6.6)	6 (3.7)	
Prostate	178 (10.9)	72 (21.5)	22 (13.5)	
Skin	36 (2.2)	7 (2.1)	1 (0.6)	
Soft tissue	28 (1.7)	2 (0.6)	2 (1.2)	
Unknown	51 (3.1)	7 (2.1)	8 (4.9)	
Insurance carrier				**<.001**
Clinical trial	15 (0.9)	2 (0.6)	4 (2.4)	
Dual Medicare/Medicaid	41 (2.5)	17 (5.1)	7 (4.3)	
Employment/individual/commercial	671 (41.1)	119 (35.5)	53 (32.5)	
Medicaid	73 (4.5)	30 (8.9)	21 (12.9)	
Medicare	550 (33.7)	110 (32.8)	56 (34.4)	
Military	41 (2.5)	15 (4.5)	5 (3.1)	
No insurance	91 (5.6)	17 (5.1)	11 (6.7)	
Other/unknown	150 (9.2)	25 (7.5)	6 (3.7)	
Treatment intent				**<.001**
Palliative	298 (18.3)	35 (10.4)	9 (5.5)	
Curative	1,299 (79.6)	293 (87.5)	146 (89.6)	
Unknown	35 (2.1)	7 (2.1)	8 (4.9)	
Distance to radiotherapy facility, miles				.231
0.00-5.00	186 (11.4)	42 (12.5)	27 (16.5)	
5.01-25.00	1,021 (62.6)	216 (64.5)	101 (62.0)	
>25.00	425 (26.0)	48 (23.0)	35 (21.5)	
Daily prescribed fraction size, cGy				**<.001**
≤225	1,007 (61.7)	245 (73.1)	145 (89.0)	
>225	625 (38.3)	90 (26.9)	18 (11.0)	
Total prescribed treatment dose, cGy				**<.001**
<5,000	478 (29.3)	58 (17.4)	20 (12.3)	
5,000-5,999	523 (32.0)	81 (24.2)	44 (27.0)	
≥6,000	631 (38.7)	195 (58.4)	99 (60.7)	
No. of prescribed days				**<.001**
<25	707 (43.3)	83 (24.8)	18 (11.0)	
≥25	925 (56.7)	252 (75.2)	145 (89.0)	
Social vulnerability level at census tract level				**.021**
Very low	486 (29.8)	92 (27.5)	53 (32.5)	
Low	538 (33.0)	105 (31.3)	39 (23.9)	
Moderate	433 (26.5)	103 (30.8)	41 (25.2)	
High	175 (10.7)	35 (10.4)	30 (18.4)	

NOTE. Bold numbers represent statistically significant at .05 *P* value.

Abbreviation: RT, radiation therapy.

### Multivariate Modeling of RTI Risk Associations

We used multinomial logistic regression analysis (Table 3) to model associations between patient characteristics and missed radiation treatment days, categorized either as moderate (2-4 days) or severe (≥5 days).

**TABLE 3.** Multinomial Logistic Regression Modeling Predictive Variable Associations With Radiation Therapy Interruption Severity

Variable	Missing 2-4 Days *v* Missing 0-1 Days				Missing 5+ Days *v* Missing 0-1 Day			
	COR [95% CI]	*P*	AOR [95% CI]	*P*	COR [95% CI]	*P*	AOR [95% CI]	*P*
Sex								
Female	Reference		Reference		Reference		Reference	
Male	**1.44 [1.14 to 1.18]**	**.003**	0.88 [0.62 to 1.24]	.467	1.34 [0.98 to 1.86]	.068	0.88 [0.55 to 1.42]	.610
Age, years								
<50	Reference		Reference		Reference		Reference	
50-65	1.38 [0.90 to 2.11]	.137	1.22 [0.77 to 1.92]	.393	1.10 [0.65 to 1.87]	.713	1.01 [0.56 to 1.84]	.967
>65	1.51 [1.01 to 2.28]	.048	1.31 [0.80 to 2.15]	.286	1.03 [0.62 to 1.72]	.909	0.84 [0.43 to 1.64]	.615
Race								
White	Reference		Reference		Reference		Reference	
Black or others	1.14 [0.71 to 1.83]	.581	1.01 [0.60 to 1.68]	.998	1.23 [0.66 to 2.29]	.513	0.81 [0.39 to 1.65]	.553
Marital status								
Single	1.14 [0.82 to 1.58]	.432	0.99 [0.69 to 1.44]	.978	1.41 [0.91 to 2.18]	.126	1.06 [0.63 to 1.78]	.834
Married	Reference		Reference		Reference		Reference	
Separated/divorced	**1.41 [1.03 to 1.91]**	**.030**	1.38 [0.99 to 1.94]	.061	**2.04 [1.38 to 3.03]**	**<.001**	**1.86 [1.18 to 2.94]**	**.007**
Unknown	1.18 [0.77 to 1.82]	.448	1.27 [0.81 to 2.01]	.296	1.04 [0.54 to 2.00]	.914	1.13 [0.56 to 2.28]	.723
Primary disease site								
Benign	1.41 [0.60 to 3.32]	.428	1.44 [0.58 to 3.57]	.429	1.82 [0.51 to 6.42]	.353	1.59 [0.42 to 6.01]	.497
Breast	Reference		Reference		Reference		Reference	
CNS	0.78 [0.37 to 1.62]	.501	0.77 [0.34 to 1.73]	.531	0.78 [0.23 to 2.67]	.687	0.59 [0.15 to 2.30]	.447
GI	1.35 [0.83 to 2.21]	.229	1.33 [0.73 to 2.44]	.531	**2.56 [1.30 to 5.02]**	**.007**	1.95 [0.85 to 4.51]	.117
Genitourinary	**3.53 [1.28 to 9.76]**	**.015**	**3.81 [1.24 to 11.66]**	**.019**	**17.65 [6.82 to 45.72]**	**<.001**	**15.62 [4.94 to 49.45]**	**<.001**
Gynecologic	1.21 [0.64 to 2.26]	.558	1.05 [0.51 to 2.15]	.901	**4.65 [2.36 to 9.17]**	**<.001**	**2.97 [1.30 to 6.79]**	**.010**
Head and neck	1.80 [1.19 to 2.74]	.005	1.28 [0.73 to 2.26]	.388	**4.17 [2.36 to 7.38]**	**<.001**	**2.31 [1.10 to 4.87]**	**.027**
Hematologic	0.74 [0.28 to 1.92]	.532	1.22 [0.42 to 3.50]	.714				
Lung/thoracic	2.07 [1.36 to 3.15]	.001	1.49 [0.87 to 2.56]	.148	**2.03 [1.03 to 4.01]**	**.042**	1.06 [0.47 to 2.39]	.896
Metastasis	0.97 [0.58 to 1.64]	.914	1.54 [0.76 to 3.13]	.714	0.79 [0.31 to 2.02]	.629	1.73 [0.54 to 5.50]	.355
Prostate	**2.86 [1.95 to 4.20]**	**<.001**	**2.41 [1.34 to 4.40]**	**.004**	**2.62 [1.03 to 4.92]**	**.003**	2.06 [0.85 to 4.98]	.107
Skin	1.37 [0.56 to 3.23]	.466	1.08 [0.43 to 2.72]	.875	0.59 [0.08 to 4.52]	.610	0.36 [0.04 to 2.95]	.343
Soft tissue	0.50 [0.12 to 2.17]	.358	0.48 [0.10 to 2.20]	.346	1.51 [0.34 to 6.81]	.588	1.02 [0.19 to 5.45]	.980
Unknown	0.97 [0.42 to 2.24]	.943	0.82 [0.25 to 2.68]	.746	**3.33 [1.39 to 7.94]**	**.007**	2.23 [0.58 to 8.48]	.244
Insurance carrier								
Clinical trials	0.75 [0.17 to 3.33]	.707	0.65 [0.14 to 2.96]	.577	**3.38 [1.08 to 10.53]**	**.036**	3.15 [0.86 to 11.46]	.082
Dual coverage	2.34 [1.29 to 4.25]	.005	1.83 [0.95 to 3.54]	.070	2.16 [0.92 to 5.05]	.075	1.71 [0.66 to 4.42]	.266
Employment/individual/commercial	Reference		Reference		Reference		Reference	
Medicaid	**2.32 [1.45 to 3.70]**	**<.001**	**2.22 [1.32 to 3.73]**	**.003**	**3.64 [2.08 to 6.38]**	**.001**	**3.43 [1.77 to 6.64]**	**<.001**
Medicare	1.13 [0.85 to 1.49]	.405	0.96 [0.69 to 1.35]	.835	1.29 [0.87 to 1.91]	.204	1.44 [0.88 to 2.34]	.146
Military	2.06 [1.11 to 3.84]	.023	1.65 [0.85 to 3.20]	.138	1.54 [0.59 to 4.07]	.380	1.37 [0.48 to 3.89]	.551
No insurance	1.05 [0.61 to 1.83]	.854	1.06 [0.59 to 1.89]	.844	1.53 [0.77 to 3.04]	.224	1.32 [0.63 to 2.74]	.458
Other insurance	0.94 [0.60 to 1.50]	.794	0.95 [0.59 to 1.53]	.825	0.51 [0.21 to 1.20]	.122	0.50 [0.21 to 1.21]	.125
Distance to radiotherapy facility, miles								
0.00-5.00	1.25 [0.82 to 1.88]	.297	1.22 [0.78 to 1.93]	.384	**1.76 [1.04 to 2.99]**	**.036**	1.76 [0.95 to 3.23]	.070
5.01-25.00	1.17 [0.88 to 1.55]	.284	1.29 [0.92 to 1.80]	.135	1.20 [0.80 to 1.79]	.370	1.42 [0.88 to 2.29]	.145
≥25.00	Reference		Reference	.312	Reference			
Daily treatment dose, cGy								
≤225	**1.69 [1.30 to 2.19]**	**<.001**	1.23 [0.82 to 1.86]	.312	**5.00 [3.03 to 8.24]**	**<.001**	**2.55 [1.21 to 5.37]**	**.014**
>225	Reference		Reference		Reference		Reference	
Total prescribed treatment dose, cGy								
<5,000	Reference		Reference		Reference		Reference	
5,000-5,999	**0.39 [0.28 to 0.54]**	**<.001**	1.29 [0.79 to 2.11]	.307	**0.27 [0.16 to 0.44]**	**<.001**	1.80 [0.89 to 3.66]	.100
≥6,000	**0.50 [0.38 to 0.67]**	**<.001**	1.65 [0.99 to 2.76]	.057	**0.54 [0.37 to 0.78]**	**.001**	**2.30 [1.09 to 4.88]**	**.029**
No. of prescribed days								
<25	Reference		Reference		Reference		Reference	
≥25	**2.32 [1.78 to 3.03]**	**<.001**	1.24 [0.76 to 2.03]	.380	**6.16 [3.74 to 10.15]**	**<.001**	1.84 [0.82 to 4.13]	.137
Treatment intent								
Curative	Reference		Reference		Reference		Reference	
Palliative	0.52 [0.36 to 0.76]	.001	0.89 [0.47 to 1.70]	.732	**0.27 [0.14 to 0.53]**	**<.001**	1.32 [0.44 to 3.98]	.621
Unknown	0.89 [0.39 to 2.02]	.774	1.33 [0.42 to 4.19]	.629	2.03 [0.93 to 4.47]	.077	1.83 [0.51 to 6.53]	.350
Smoking status								
Current smoker	**2.28 [1.50 to 3.47]**	**<.001**	1.58 [0.97 to 2.57]	.063	**2.91 [1.68 to 5.04]**	<.001	1.74 [0.91 to 3.34]	.095
Former smoker	1.25 [0.85 to 1.82]	.244	1.06 [0.71 to 1.59]	.776	1.27 [0.74 to 2.16]	.382	1.05 [0.59 to 1.82]	.876
Never smoke	Reference		Reference		Reference		Reference	
Unknown	1.02 [0.75 to 1.39]	.899	0.74 [0.45 to 1.19]	.216	1.08 [0.69 to 1.68]	.734	1.07 [0.56 to 2.02]	.841
Substance abuse								
Current	1.69 [0.86 to 3.32]	.125	1.18 [0.56 to 2.49]	.654	2.50 [1.17 to 5.35]	.019	1.50 [0.62 to 3.66]	.372
Denies	Reference		Reference		Reference		Reference	
Past	**2.64 [1.33 to 5.26]**	**.006**	2.01 [0.93 to 4.35]	.075	1.60 [0.54 to 4.69]	.394	0.85 [0.26 to 2.82]	.794
Unknown	1.01 [0.79 to 1.29]	.967	1.25 [0.47 to 3.38]	.656	0.89 [0.63 to 1.26]	.511	2.20 [0.74 to 6.54]	.157
Alcohol use								
Current	0.99 [0.72 to 1.37]	.980	0.97 [0.69 to 1.36]	.868	0.75 [0.49 to 1.16]	.198	0.80 [0.50 to 1.28]	.351
Denies	Reference		Reference		Reference		Reference	
Past	1.10 [0.64 to 1.88]	.737	0.98 [0.55 to 1.77]	.955	0.78 [0.36 to 1.68]	.522	0.70 [0.30 to 1.65]	.420
Unknown	0.95 [0.72 to 1.26]	.719	1.12 [0.40 to 3.12]	.825	0.69 [0.47 to 1.01]	.053	0.42 [0.13 to 1.39]	.157
Social vulnerability level								
Low	Reference		Reference		Reference		Reference	
Very low	0.97 [0.71 to 1.32]	.845	0.95 [0.66 to 1.35]	.759	1.50 [0.98 to 2.32]	.063	1.49 [0.89 to 2.48]	.130
Moderate	1.22 [0.90 to 1.64]	.196	1.20 [0.86 to 1.69]	.283	1.31 [0.83 to 2.06]	.251	1.31 [0.77 to 2.22]	.320
High	1.02 [0.67 to 1.56]	.909	0.90 [0.54 to 1.49]	.677	**2.36 [1.43 to 3.92]**	**.001**	**2.60 [1.32 to 5.09]**	**.005**

NOTE. Bold numbers represent statistically significant at .05 *P* value.

Abbreviations: AOR, adjusted odds ratio; COR, crude odds ratio.

#### Moderate Treatment Interruption (2-4 treatment days)

Adjusted regression analysis identified significant associations between cancer disease site and insurance type with moderate RTI. Patients with genitourinary cancer had 3.81 times higher odds of moderate delay compared with patients with breast cancer (AOR, 3.81; 95% CI, 1.24 to 11.66). Similarly, patients with prostate cancer had 2.44 times higher odds of moderate delay compared with patients with breast cancer (AOR, 2.44; 95% CI, 1.34 to 4.46). Medicaid beneficiaries had 2.22 times higher odds of moderate delay compared with those with employer-sponsored/individual/commercial insurance (AOR, 2.22; 95% CI, 1.32 to 3.73).

#### Severe Treatment Interruption (5 or more treatment days)

Marital status, treatment sites, insurance type, daily effective fraction dose, total prescribed dose, and social vulnerability level were significantly associated with severe delay.

Divorced or separated patients (AOR, 1.86; 95% CI, 1.18 to 2.94) had higher odds of severe delay relative to married patients. Patients with gynecologic cancer (AOR, 2.97; 95% CI, 1.30 to 6.79), head and neck cancer (AOR, 2.31; 95% CI, 1.10 to 4.87), and genitourinary cancer (AOR, 15.62; 95% CI, 4.94 to 49.45) experienced higher risk for severe delays compared with patients with breast cancer. Medicaid coverage (AOR, 3.43; 95% CI, 1.77 to 6.64) conferred higher risk relative to individual, commercial, or employment-based insurance. Patients prescribed a daily dose of ≤225 cGy (AOR, 2.55; 95% CI, 1.21 to 5.37) or were prescribed a total dose of ≥6,000 cGy (AOR, 2.30; 95% CI, 1.09 to 4.88) were at higher risk. Finally, patient home residence in neighborhoods with high SVI (AOR, 2.60; 95% CI, 1.32 to 5.09) was significantly associated with severe treatment delays.

### Geospatial Distribution of RTI

**FIG 2.**
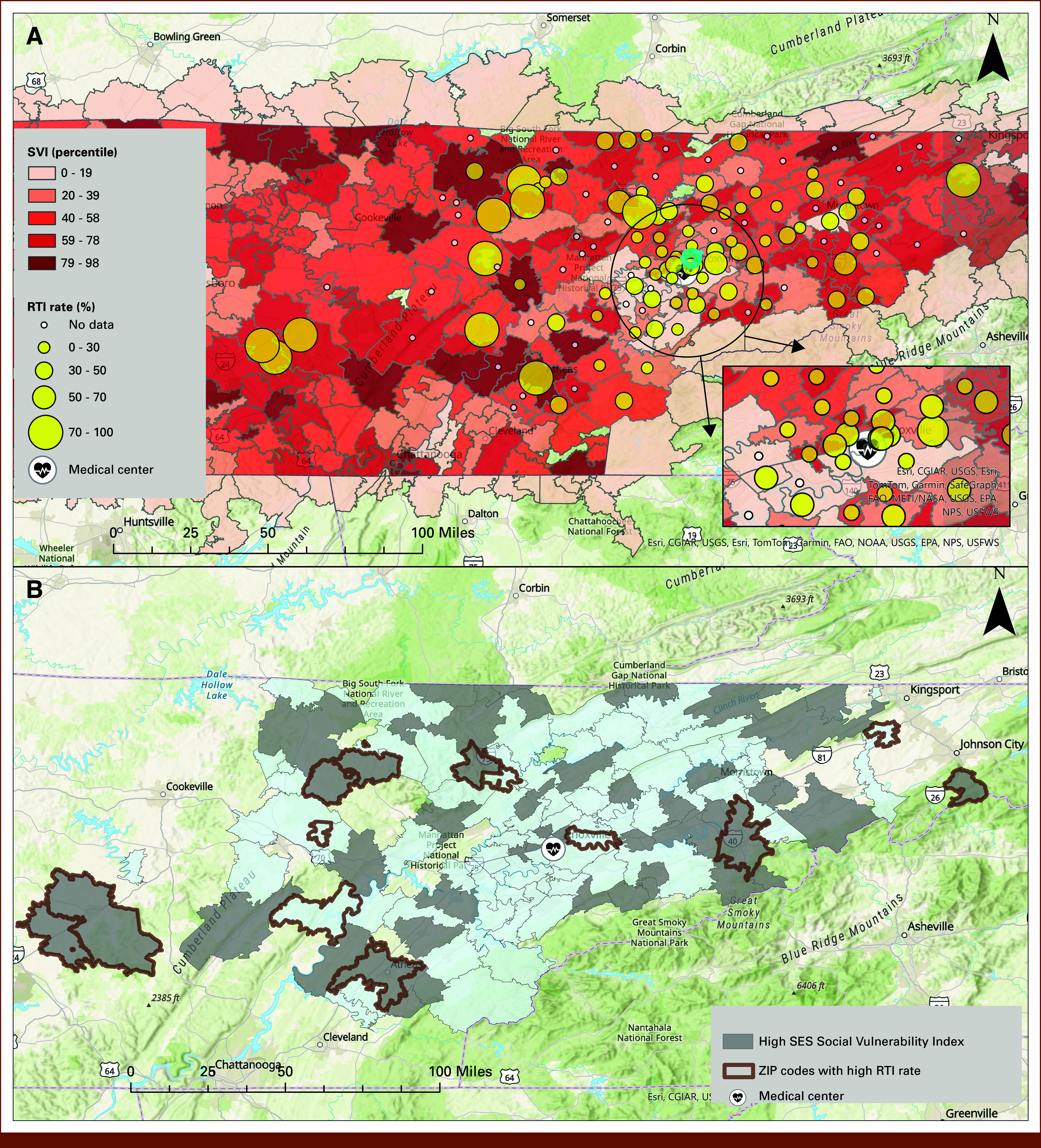
ZIP code–level analysis of RTI rates in the study area (2016-2022). (A) Spatial distribution of RTI rates represented by yellow circles, overlaid on the SVI by ZIP code. (B) Geographic overlap of ZIP codes with both high social vulnerability (SVI > 0.5 percentile) and high RTI rates (≥50%). RTI, radiation therapy interruptions; SES, socioeconomic status; SVI, Social Vulnerability Index.

Figure 2 presents ZIP code–level variation in radiation therapy RTI rates across the geographic catchment region. In Figure 2A, RTI rates are represented by the size of yellow circles overlaid on ZIP codes shaded by SVI; darker red areas indicate higher social vulnerability. The inset image shows the Knoxville, TN metropolitan region in greater detail. Most ZIP codes experiencing the highest interruption rates are located outside Knoxville's urban core, particularly in socially vulnerable regions. Figure 2B highlights ZIP codes with both high SVI (above the 50th percentile) and high RTI rates (≥50%), outlined in brown. While Figure 2A focuses on illustrating the overall geographic variability in RTI rates relative to SVI, Figure 2B emphasizes ZIP codes where elevated social vulnerability and high RTI rates coincide, highlighting areas of particular concern that warrant targeted intervention.

## DISCUSSION

This report demonstrates that close to one fourth of radiotherapy patients in our Appalachian catchment area miss two or more days of scheduled treatment, with nearly 10% missing a week or longer. This closely tracks published findings observed in the socially and geographically distinct Mississippi River Delta region of Tennessee, where rates of minor and major RTI were 25.8 and 9%, respectively.^11^ The clinical impact of such interruptions on local control and cure are well documented, with a delay of even a single treatment resulting in as much as a 1.4% decrease in tumor control, and up to a 20% reduction with missing seven or more days in head and neck treatment.^17^

Our study identifies multiple factors associated with treatment interruption. These include specific primary cancer site diagnoses such as head and neck, gynecologic, and genitourinary cancers, as well as socioeconomic factors including Medicaid coverage, marital status, and socially vulnerable neighborhood locations, similar to previous findings.^11^ Specific cancer sites such as GI, head and neck, and gynecologic cancers are often treated with protracted radiation schedules and multiple forms of treatment, making them more susceptible to treatment interruptions. Additionally, greater side effects can result from radiation treatment to certain sites, such as head and neck cancer, making treatment adherence differentially more challenging. By contrast, breast cancer (the reference standard for our analysis) is typically treated over a shorter period of time and is well tolerated. Although many of these static features are not modifiable, identifying patients at elevated risk for RTI creates opportunities to deliver high-yield, personalized support interventions to individuals at greatest need. This is a radiotherapy population for which assistance with transportation, health literacy–appropriate education, and/or enhanced navigational support could have an outsized impact on treatment delivery quality and cancer control outcomes. Our group's ongoing work continues to focus on the development and deployment of such interventions in a risk-informed manner.

Although many of the factors associated with RTI are patient-specific, it is worth noting that a physician-defined factor, traditional daily treatment fractionation, is associated with a 2.55 times risk for severe RTI. Although currently accepted use of hypofractionated therapy varies across cancer site diagnoses, these data suggest that its implementation could benefit treatment quality while simultaneously providing potential cost-savings and increased patient convenience.

Use of a state-wide data warehouse facilitated integration of health records, radiation treatment information, and neighborhood-level SDH. Many patient-level data elements were initially unstructured and not ready for direct modeling, requiring substantial preprocessing efforts. Leveraging large language model (LLM)–based data preprocessing allowed us to more efficiently standardize categorization of these unstructured data elements, particularly for insurance carrier type and ICD-10–based diagnostic coding. We have formally compared open-source LLM model accuracy in previous preparatory work for this project (manuscript submitted), where GPT-4.0 and BioBERT achieved over 90% accuracy for ICD-coded diagnoses and over 81% accuracy for free-text entries, with classifications validated by oncology experts. Generative AI tools have shown potential to streamline data preprocessing in large-scale clinical and population health studies, reducing manual effort and enhancing data quality for subsequent analyses.^18^

In the current analysis, travel distance was not significantly associated with RTI in our final model. However, we have observed similar findings in previously published analysis of H&N cancer cases in SEER registry data.^19^ Increased travel distance from rural areas for specialized care has repeatedly been shown to not negatively affect time to initiation of treatment. This type of relationship has been termed the urban-rural paradox by Haggerty et al,^20^ who found that urban residents cited distance as a barrier to care more frequently than rural residents. Possible explanations for this phenomenon include more time spent planning for appointments, greater social and/or community support, and greater access to private transportation in rural areas.

Geographic Information System (GIS)–informed findings illustrated in Figure 2 highlight explanatory insights made possible by adding geographic context to cohort-level statistical analysis. Although our baseline multivariate modeling found no association between travel milage and RTI, Figure 2A suggests that RTI rates potentially peak in underresourced rural areas located at distance from urban treatment facilities. Likewise, Figure 2B documents geographic overlap between socially vulnerable regions and ZIP codes affected by higher RTI rates. This insight offers an opportunity for patient risk stratification on the basis of accessible home address information. Automated GIS analytic strategies, therefore, promise to inform corrective care delivery interventions and policies tailored to individual patients and neighborhoods, while retaining relevance to routine statistical analysis and higher-dimensional AI-based techniques. Of note, we performed additional exploratory investigation, which detected no RTI rate variation by rurality classification; approximately half (12 of 23) of high-RTI areas were coded as large metropolitan, while the other half were designated as small town or rural areas on the basis of US Department of Agriculture's Rural-Urban Commuting Area nomenclature.^21^ This underscores the importance of care barriers that unite urban and rural communities in common interest, a finding we have previously observed in a national head and neck cancer registry population.^19^

Limitations of this study merit discussion. First, geographic relevance of our findings is limited to the Southeastern United States. Consistent with our Appalachian patient population, the majority (approximately 94%) of our study participants who received radiotherapy were White, with only 6% belonging to minoritized populations. Future studies are currently planned in collaboration with centers across the state of Tennessee to improve generalizability of study findings across the state's extremely heterogeneous regions, which could make our regional findings and potential intervention strategies more relevant to other regions. Another limitation is the study's retrospective design; although significant correlations are observed, which make sense mechanistically, direct causative factors cannot be determined. It was not possible to determine specific causes for individual interruption events such as transportation barriers, illness, or hospitalization, all of which carry different clinical and operational implications. Finally, although neighborhood-level social determinants were examined at the census tract level, ZIP code–level aggregation was used for GIS analysis because of limited sample size at the tract level. Importantly, although the spatial analysis maps illustrate ZIP code–level associations between RTI and the SVI, these results do not account for potential confounding variables.

In conclusion, this study confirmed significant, potentially modifiable treatment interruption risks affecting radiation treatment quality in the Central Appalachian region, reproducing previous findings from the Upper Mississippi Delta region.^11^ We identified clinical and socioecological factors uniquely associated with RTI in this population; these included primary cancer site diagnosis, behavioral risks, insurance coverage, and neighborhood-level social vulnerability. Importantly, physician-dependent process of care determinants, such as use of accelerated treatment, could play a potential protective effect. Centralized electronic health data warehouses and advanced analytics (such as novel use of LLMs) promise to enhance real-time care quality feedback. Our ongoing work is guided by the hypothesis that individualized RTI risk assessment can inform design and delivery of context-specific social interventions to improve care access. Our group is currently evaluating this prospectively in an ongoing clinical trial. This study reinforces the potential need and clinical promise of this approach across Southeastern US regions.

## Data Availability

The data that support the findings of this study are available upon reasonable requests from the corresponding authors. The data set is not publicly available because of institutional privacy policies.
